# Magnetic resonance enterography, colonoscopy, and fecal calprotectin correlate in colonic Crohn’s disease

**DOI:** 10.1186/s12876-019-1125-7

**Published:** 2019-12-05

**Authors:** Alexander S. Somwaru, Vikesh Khanijow, Venkat S. Katabathina

**Affiliations:** 1 Department of Diagnostic, Molecular, and Interventional Radiology, Icahn School of Medicine at Mount Sinai, West, 1000 10th Avenue, New York, NY 10019 USA; 2 Gastroenterology, Gastro Florida, Mease Countryside Hospital, Tampa, Florida USA; 3 Department of Radiology, University of Texas Health Science Center San Antonio, San Antonio, TX USA

**Keywords:** Fecal calprotectin, Magnetic resonance enterography, Colonoscopy, Crohn’s disease

## Abstract

**Background:**

Fecal calprotectin (FCP), magnetic resonance enterography (MRE), and colonoscopy are complementary biometric tests that are used to assess patients with Crohn’s Disease (CD). While prior studies have evaluated the association between combinations of these tests, no study has established a correlation between all three: FCP, MRE, and colonoscopy. We prospectively investigated if there is correlation between these three tests, which may result in improved clinical outcomes that can then be used to streamline patient monitoring and treatment modification.

**Methods:**

One hundred fifty-six patients with colonic CD were prospectively examined between March 2017 and December 2018. FCP levels, MRE, and colonoscopy were assessed in parallel on all 156 patients. Clinical CD activity was measured with the Crohn’s Disease Activity Index (CDAI). CD activity with FCP was measured with a quantitative immunoassay. CD activity on MRE was measured with the Magnetic Resonance Index of Activity (MaRIA). CD activity on colonoscopy was measured with the Crohn’s Disease Endoscopic Index of Severity (CDEIS).

**Results:**

One hundred twelve patients (72%) had active disease (Crohn’s Disease Activity Index > 150) and 44 patients (28%) were in clinical remission disease (Crohn’s Disease Activity Index < 150). FCP levels, MaRIA, and CDEIS are highly correlated with positive and significant Pearson and Spearman coefficients, respectively (*P* < 0.0001), in univariate analyses. Regression analysis (multivariate analyses) demonstrates significant, positive correlation between FCP and MaRIA (*r* = 1.07, *P* < 0.0001) and between FCP and CDEIS (*r* = 0.71, *P* = 0.03), and between.

MaRIA and CDEIS (*r* = 0.63, *P* = 0.01).

**Conclusions:**

FCP levels significantly correlate with the degree of active inflammation in patients with colonic Crohn’s Disease. Improved clinical results may be achieved by using a biometric strategy that incorporates FCP, colonoscopy, and MRE together. This strategy may in-turn be used in the future to streamline monitoring disease activity and adjustment of therapy to improve long term patient outcomes.

## Background

Crohn’s Disease (CD) is a discontinuous transmural inflammatory disease that can involve the whole gastrointestinal tract and it is part of inflammatory bowel disease group. CD is an evolving disease and severity of inflammation and disease location may change [1–5]. Periodic monitoring of patients with CD is therefore crucial in the management of the disease. The method and frequency of monitoring varies upon patients’ symptoms, different degrees of disease severity, and how patients’ respond to pharmacologic therapy [1]. In this investigation, we explore the correlation between three non-invasive and invasive tests: FCP, colonoscopy, and MRE.

FCP is a non-invasive test that uses as a biomarker of inflammation to detect and monitor Crohn’s Disease (CD) activity [1, 2]. The biomarker, FCP, is a heat stable granulocyte-derived protein that is released by activated neutrophils of the intestinal immune system in response to inflammation and then absorbed into feces [2]. Numerous studies have shown that FCP levels correlate well with intestinal inflammation with high levels of sensitivity and specificity [6–11]. Colombel et al., in one of the largest trials of tight control management of patients’ with CD, established a FCP level of 250 μg/g or greater as abnormally elevated [12].

Colonoscopy plays a fundamental role in the diagnosis and monitoring of patients with CD. This technique enables both diagnostic analysis, such as direct visualization of the mucosa and histologic examination [3–5]. The Crohn’s Disease Endoscopic Index of Severity (CDEIS) is an established system that is used to measure severity and extent of disease seen on colonoscopy [3–5].

MRE is a non-invasive imaging technique used to both diagnose and assess disease activity in patients with CD as well as an array of infectious and neoplastic disorders of the gastrointestinal tract [5, 7, 13]. MRE uses dynamic, high spatial resolution and soft tissue characterization of the bowel to provide vital anatomic and physiologic information without exposing patients to unnecessary ionizing radiation [5–7, 13]. Rimola et al. established MaRIA as the first and validated radiological classification system used to quantitatively measure severity and extent of disease on MRE that has correlated well with colonoscopy and CDEIS [5].

A correlation between all three tests: FCP, MRE, and colonoscopy, has to our knowledge, never been shown in the same cohort of patients with colonic CD. The aim of this prospective investigation is to determine statistically if FCP levels correlate with validated MRE and colonoscopic scoring systems: MaRIA and CDEIS, respectively.

## Methods

### Patient selection

This study was performed in compliance with the 1996 Health Information Portability and Accountability Act (HIPAA). The investigation location and source of the participants was MedStar Georgetown University Hospital in Washington, D.C., where institutional review board granted approval and all patients provided written informed consent to participate in this prospective study. A standardized research protocol for the data collection was utilized.

A total of 156 consecutive patients were enrolled in this study. Inclusion criteria were informed consent, 18 years of age or older, known diagnosis of colonic CD, MRE performance, measurement of FCP levels within a maximum of two weeks prior to MRE, colonoscopy within a maximum of two weeks before or two weeks after the MRE, and no pharmacological therapy modification. Exclusion criteria were age younger than 18 years, no diagnosis of small bowel CD or small bowel and colonic CD confirmed by prior ileoscopy and biopsy, intolerance or contraindication to performance of MRE (such as pacemakers, MR-incompatible hardware, severe claustrophobia, and pregnancy), colonoscopy not performed two weeks before or two weeks after MRE, and FCP measurement not within a maximum of two weeks prior to MRE. The pharmacological therapies of patients were 5-aminosalicylic acids, corticosteroids, immunosuppressants, and/or biologic agents. Pharmacological therapy was not modified between FCP level measurement, MRE, and colonoscopy.

### Crohn’s disease clinical activity

At our institution, clinical disease activity was calculated for each patient at the time of MRE with the Crohn’s Disease Activity Index (CDAI) score. A CDAI score less than 150 indicated clinically inactive disease; scores of greater than 150 indicated active disease.

### MR enterography acquisition

At our institution, MRE is performed using 3.0 Tesla magnet systems (Siemens Healthcare, Berlin, Germany). Patients ingest 1450 mL of a barium sulfate suspension (VoLumen; Bracco, Westbury, New York, U.S.A.) followed by 500 mL of water in divided doses one hour before the exam to achieve adequate bowel distention. Subsequently, a 0.5 mg dose of glucagon is administered intramuscularly prior to image acquisition to reduce bowel peristalsis. A second 0.5 mg dose of intramuscular glucagon (Eli Lilly, Indianapolis, Indiana, U.S.A.) is administered prior to the administration of intravenous gadolinium-based contrast material. Multi-planar MR imaging of the abdomen and pelvis was performed with a dedicated phased array torso coil using the following protocol (Table 1): coronal and axial T2-weighted half-Fourier acquisition single-shot turbo spin-echo (HASTE); T1-weighted dual gradient echo sequences; coronal fat-saturated T2-weighted true fast imaging with steady state precession (TrueFISP); coronal and axial T1 pre-contrast fat saturated (FS); serial dynamic coronal T1-weigted dynamic volume interpolated breath hold examination (VIBE) fat-saturated images obtained approximately 25 s, 60 s, 90 s after gadolinium contrast material (Gadavist 0.1 mmol/kg; Bayer Healthcare Pharmaceuticals, Wayne, New Jersey, U.S.A.) intravenous injection; axial b50 and axial b800 diffusion-weighted images with apparent diffusion coefficient (ADC) mapping; and axial fat-saturated T1-weighted delayed VIBE images.

**Table 1:**
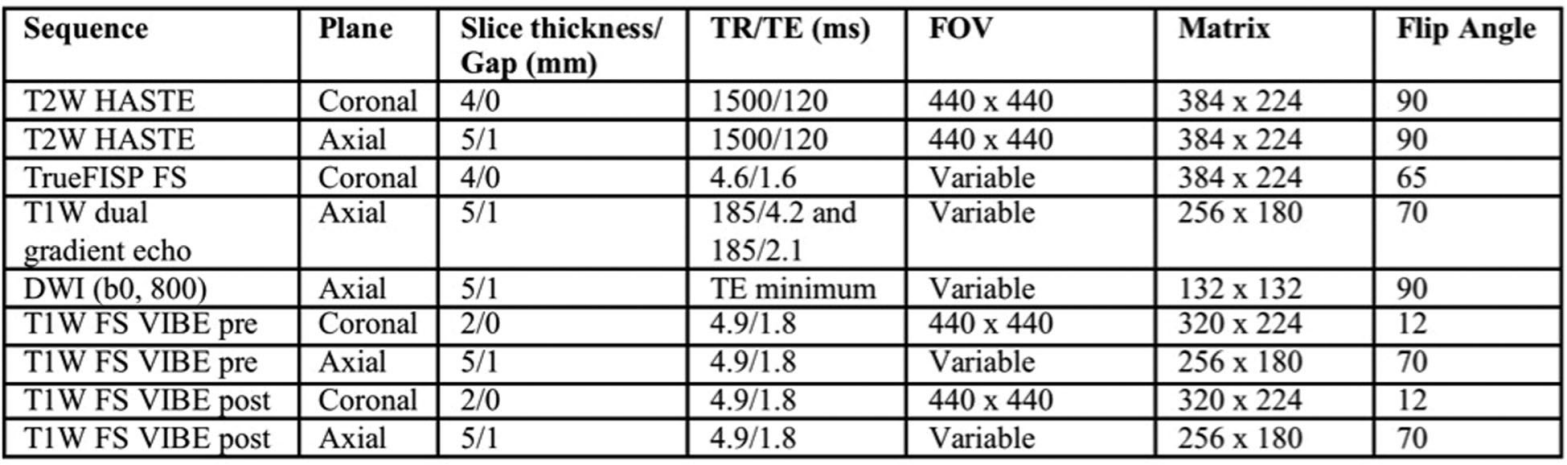
MRE Acquisition Protocol

### MR enterography analysis: MaRIA

Two abdominal radiologists with twelve and five years of experience in interpreting MRE, respectively, independently reviewed the images from each MRE exam for the pattern and extent of abnormalities. The radiologists used Picture Archiving and Communication System (PACS) (IntelliSpace 4.4, Philips Healthcare, Amsterdam, Netherlands) on two separate workstations. The radiologists were blinded to colonoscopy results, FCP levels, and clinical and laboratory data.

Imaging features of inflammation are easily shown on MRE. Wall thickness that measures greater than 3 mm is abnormal; this thickening is due to edema and inflammation, which result in slightly increased signal intensity on T2-weighted HASTE and TrueFISP images. Contrast-enhanced T1-weighted VIBE images of the inflamed and thickened bowel show patterned wall hyperenhancement (12). Diffusion-weighted images show restricted diffusion in areas of active inflammation. Both T2-weighted images (HASTE and TrueFISP) and contrast-enhanced images show linear and transmural ulceration (12). Wall thickness, edema, contrast enhancement, and ulcers are the components used to calculate the MaRIA score of disease activity.

The radiologists each calculated a MaRIA scores for each MRE. The simplified (or segmental) MaRIA score for disease activity is calculated from the formula establish by Rimola et al.: (1.5 X wall thickness) + (0.02 X RCE enhancement) + (5 X edema) + (10 X ulceration) [5]. The cutoff value for active disease is ≥7 and for severe disease is ≥11. These cutoff values have shown high accuracy for diagnosis for both active disease: receiver operating characteristic (ROC) area 0.96, sensitivity 0.92, specificity 0.92; severe disease ROC area 0.91, sensitivity 0.87, and specificity 0.87, respectively (12). The radiologists arrived at a consensus if there was any discrepancy regarding the interpretation of the images by using the two most dominant of the four MRE features that comprised the MaRIA activity score formula.

### Colonoscopic analysis: CDEIS

At our institution, a pre-procedural oral preparation is used for bowel cleansing, which is essential for adequate mucosal examination. Variable types of sedation with cardiac and oxygen monitoring are for patient comfort and depend upon the predicted difficulty of the procedure. A digital rectal exam is performed to assess for skin tags, polyps, and fistula. Two gastroenterologists, with twelve and ten years of experience with colonoscopy of patients with CD, used a high-definition colonoscope (EC-3890Li, RiCoh, Tokyo, Japan) that is introduced through the anus and advanced through the colon. The colonoscope is connected to a multichannel system for air insufflation, suction, water, video monitor, and power supply.

The gastroenterologists were aware of the patient’s diagnosis of CD but blinded to MRE results. The gastroenterologists calculated total CDEIS score for each patient by assessing for deep ulceration (no = 0, yes = 12), superficial ulceration (no = 0, yes = 6), surface involved by disease (0–10), ulcerated surface (0–10), and ulcerated or non-ulcerated stenosis (no = 0, yes = 3).

### Fecal calprotectin analysis

The concentration of fecal calprotectin was measured at an outside laboratory using the values recommended by the laboratory (Quest Diagnostics, Secaucus, New Jersey, U.S.A.) using patients’ fecal samples (40–100 mg) and a polyclonal antibody quantitative enzyme-linked immunoassay. The concentration of calprotectin in the fecal sample was calculated using the values recommended by the laboratory. Results were expressed in microgram per gram of feces. The analytical sensitivity is 6.25 μg/mL. In this laboratory, values above 250 μg/g are considered abnormally elevated and values below 50 μg/g are considered normal.

### Statistics

The Kruskal-Wallis test was performed to evaluate the association between FCP levels and MaRIA, FCP and CDEIS, and CDEIS and MaRIA. Specifically, the Kruskal-Wallis test was used to assess difference for these non-normally distributed variables. We performed multiple univariate analyses between FCP and colonoscopy, FCP and MRE, and MRE and colonoscopy and then multivariate analysis between FCP, colonoscopy, and MRE to assess if there is independent positive correlation between each pair of these biometric tests and then between all three biometric tests.

We had no missing information for the data presented in this study. We used a correlative approach and applied multivariate regression analysis to evaluate the effect of CDEIS and MaRIA on FCP levels. All statistical analysis was performed using SAS software, version 9.4 (SAS Institute Incorporated, Cary, North Carolina, U.S.A.). A probability value of *P* < 0.05 was considered to be statistically significant.

## Results

189 patients with colonic CD were initially included in the study however 33 patients were excluded because of incomplete data. The mean age of the 156 selected patients at the time of MRE was 54 years (range, 18–90 years); 86 females and 70 males; 114 patients identified as European descent, 25 patients identified as African descent, 12 patients identified as Asian descent, and 5 patients identified with various descents. FCP levels and colonoscopy results for each patient were retrieved from the electronic health record (EHR). The median time interval to complete all exams was 8 days. Assessment of clinical activity with CDAI (> 150) showed 84 patients (54%) had clinically active disease and 74 patients (44%) had clinically inactive disease at the time of MRE.

### Correlation between FCP and CDEIS

**Fig. 1:**
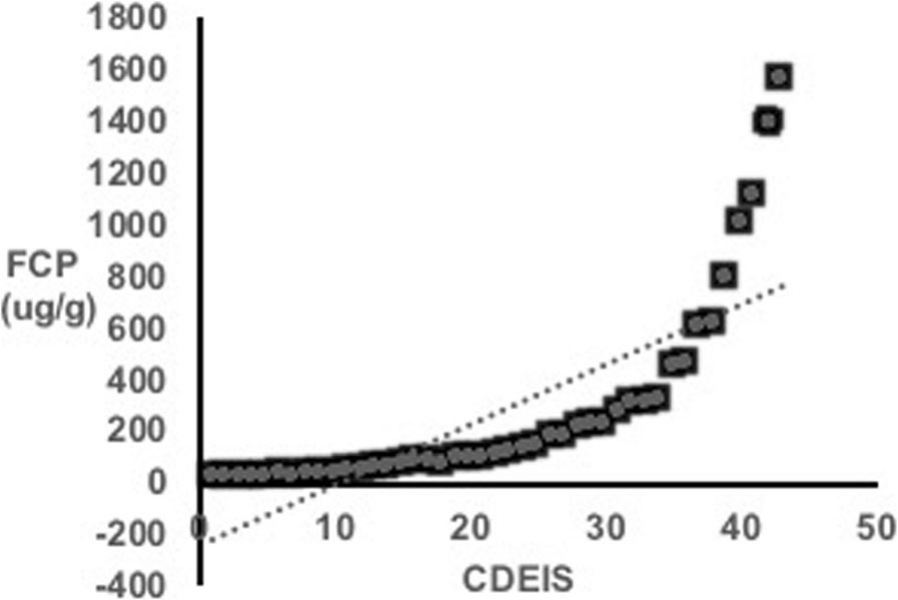
Scatter plot demonstrates the positive correlation of FCP and CDEIS from colonoscopy (Spearman’s rank correlation = 0.61; *P* < 0.001)

CD activity measured on colonoscopy with CDEIS scores showed a good relationship with FCP levels. We found a highly significant correlation between FCP levels and CDEIS scores (rho = 0.913, *P*-value < 0.0001, Fig. 1). Table 2 presents the statistical analysis between FCP and CDEIS.

**Table 2:**
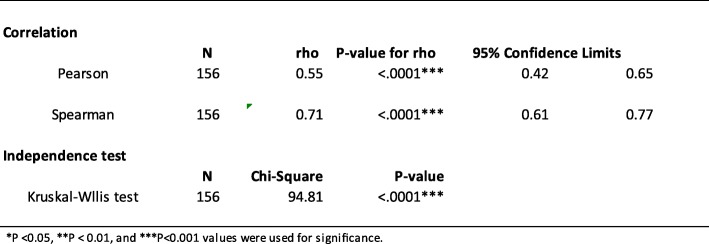
Statistical analysis of FCP and CDEIS

**Fig. 2:**
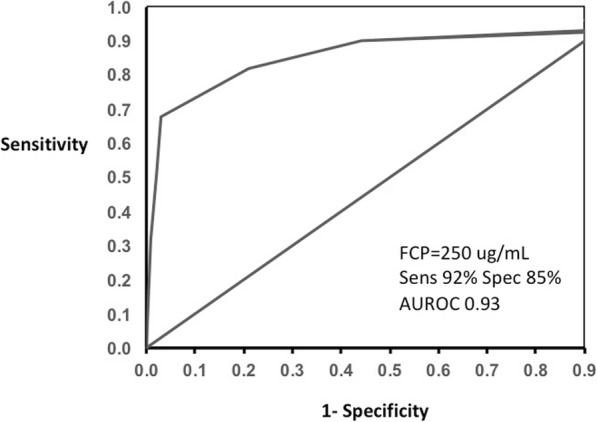
Receiver operating characteristic (ROC) curve of fecal calprotectin (FCP) values to predict active disease on colonoscopy with CDEIS. Sensitivity (Sens); specificity (Spec); area under the ROC curve (AUROC)

The *P*-values of the estimated Pearson’s (rho = 0.55) and Spearman’s (rho = 0.71) correlation coefficients were highly significant (*P* < .0001, respectively). The binomial 95% confidence intervals (CIs) were calculated for the sensitivity of FCP levels. The CIs for Pearson’s were 0.42–0.65 while for Spearman’s were 0.61–0.77. The Kruskal-Wallis chi-square test (Chi-square = 94.81, *P* < .0001) was highly significant and indicated the independence of the colonoscopy and FCP level. The results suggested that there was a statistically significant difference between the underlying distributions of FCP and CDEIS. The area under the ROC curve of 0.932 (95% CI, 0.861–0.956) confirmed our cutoff FCP value of 250 μg/mL to predict the presence of active disease on colonoscopy with CDEIS (Fig. 2). Sensitivity was 92.04% (95% CI, 83.58–97.21) and specificity was 85% (95% CI, 68.91–93.48).

### Correlation between FCP and MaRIA

CD activity measured on MRE with MaRIA scores showed a good relationship with FCP levels. The correlation between FCP levels and MaRIA scores were assessed. The median FCP levels were significantly different between patients with active disease (CMDI > 9) and without active disease (MaRIA < 7) on MRE using the Mann-Whitney U test (765.5 μg/g versus 98 μg/g, *P* < 0.01).

The *P*-values of the estimated Pearson’s (rho = 0.58, *P* < .0001) and Spearman’s (rho = 0.71, *P* < .0001) were highly significant. The CI’s for Pearson’s were 0.46–0.67 while for Spearman’s were 0.62–0.78 (Table 3). Testing for independence of colonoscopy and MRE the Kruskal-Wallis chi-square test (65.84) was highly significant (*P*-value <.0001) between MRE levels and colonoscopy (Table 3).

**Table 3:**
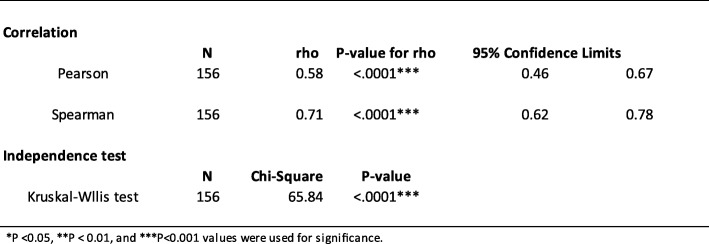
Statistical analysis of FCP and MaRIA

**Fig. 3:**
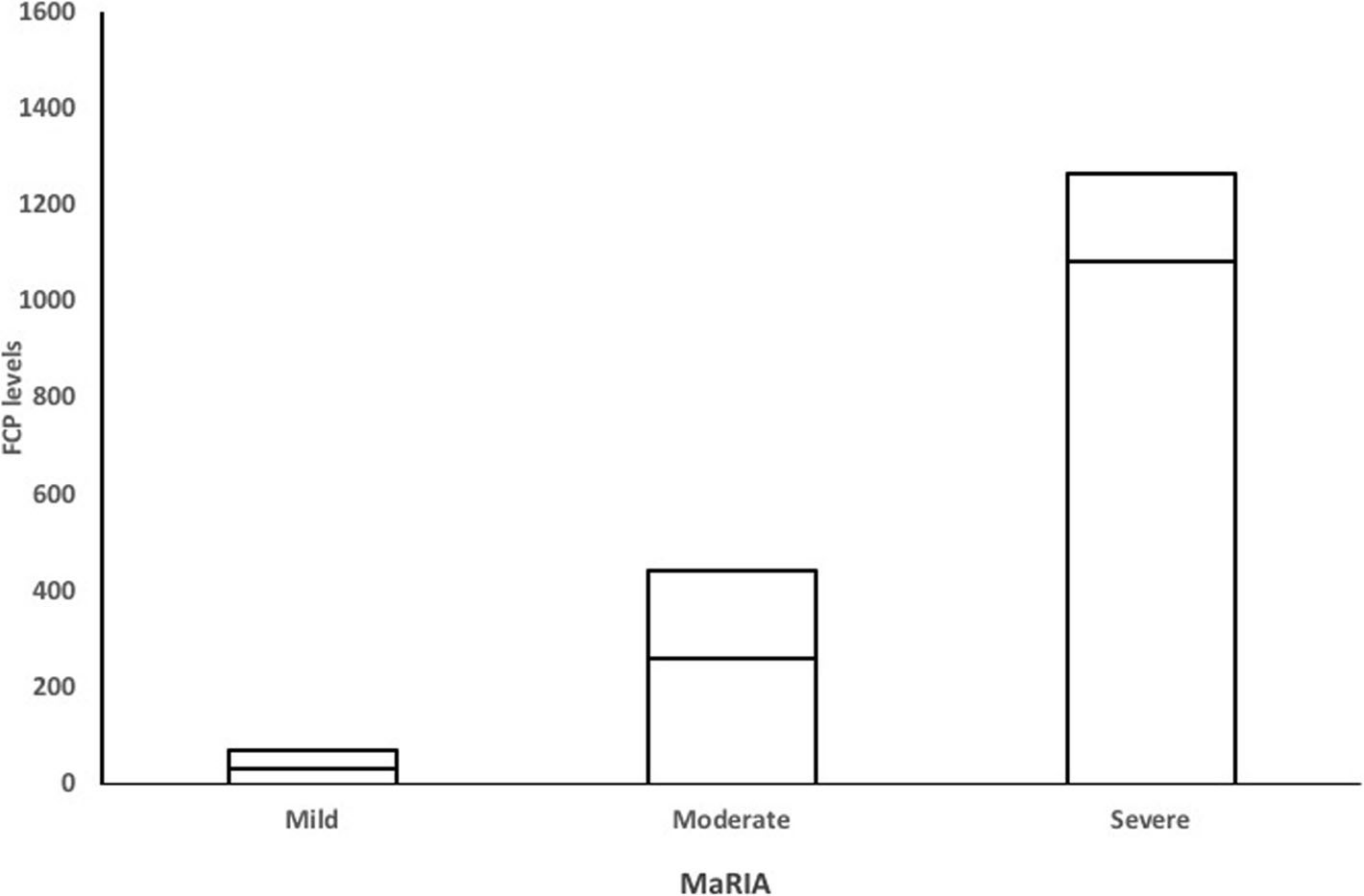
FCP levels in different severity levels and MaRIA grades. The horizontal line in the middle of the box is the median while the box represents the upper and lower quartiles

**Fig. 4:**
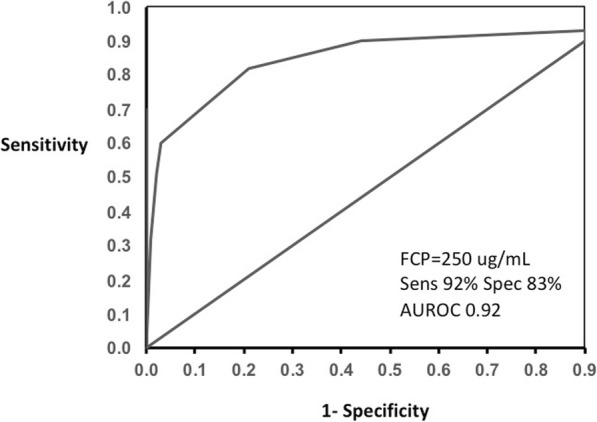
Receiver operating characteristic (ROC) curve of fecal calprotectin (FCP) values to predict active disease on MRE with MaRIA. Sensitivity (Sens); specificity (Spec); area under the ROC curve (AUROC)

MaRIA grades (active, severe) are significantly different and the median levels of FCP are elevated with the severity of inflammation on MRE (Fig. 3). The area under the ROC curve of 0.922 (95% CI, 0.874–0.957) confirmed our cutoff FCP value of 250 μg/mL to predict active disease on MRE with MaRIA (Fig. 4). Sensitivity was 92.04% (95% CI, 83.58–97.21) and specificity was 83% (95% CI, 67.91–92.31).

### Correlation between CDEIS and MaRIA

Finally, the correlation between CDEIS scores and MaRIA were assessed and also showed a good relationship. The *P*-values of the estimated Pearson’s (rho = 0.71, *P* < .0001) and Spearman’s (rho = 0.49, *P* < .0001) were highly significant. The CIs for Pearson’s were 0.61–0.77 while for Spearman’s were 0.37–0.61 (Table 4). Testing for independence of colonoscopy and MRE the Kruskal-Wallis chi-square test (121.82) was highly significant (*P*-value <.0001) between MRE levels and colonoscopy (Table 4). The median CDEIS was 22 with a range of 6 to 38; median MaRIA was 22.5 with a range of 5 to 30.

**Table 4:**
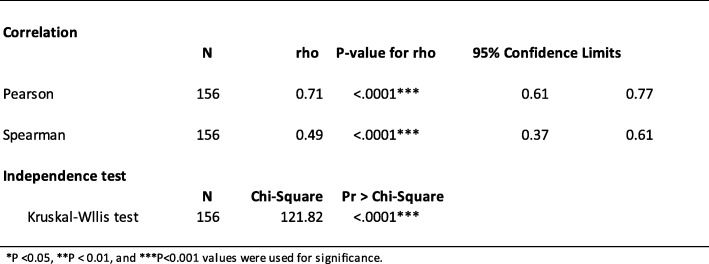
Statistical analysis of CDEIS and MaRIA

### Multivariate analysis between FCP, MaRIA, and CDEIS

**Fig. 5:**
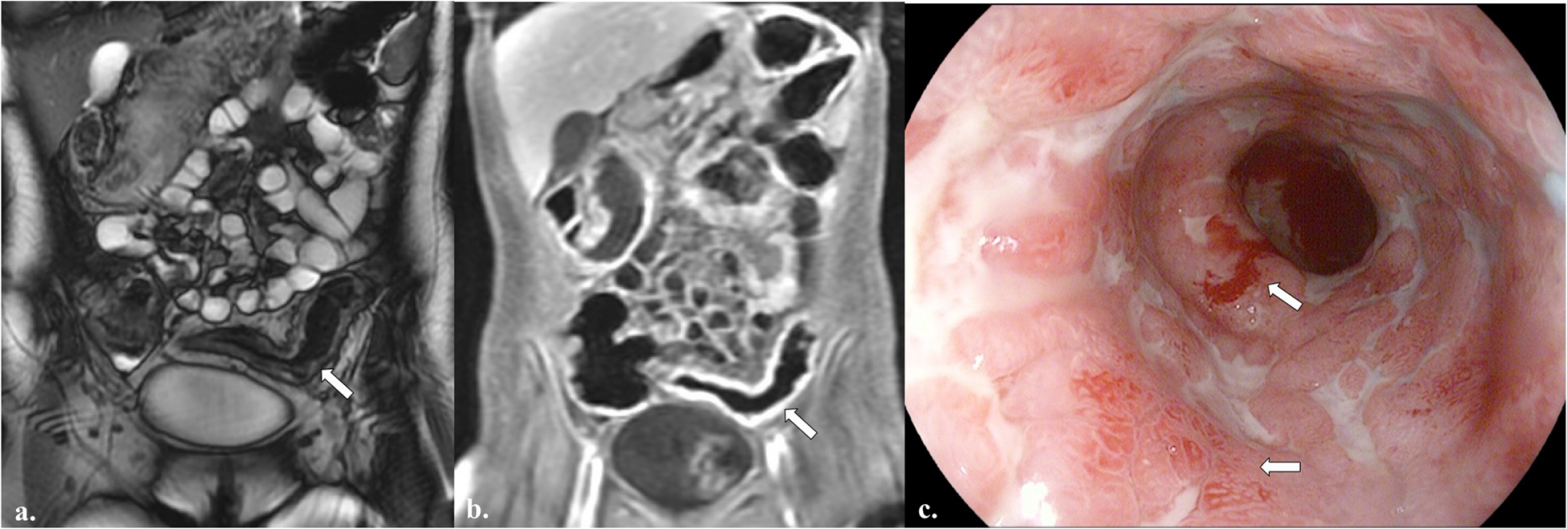
Correlation between FCP, MaRIA, and CDEIS. A 27 year-old female with colonic CD experienced abdominal pain of increasing severity. The patient had an elevated FCP level of 436 μg/g, CDEIS of 26 on colonoscopy, and MaRIA score of 15 on MRE, which corresponds with a grade of severe. **a.** Coronal fat-saturated T2-weighted true fast imaging with steady state precession (TrueFISP) image from MRE shows circumferential wall thickening and edema in the sigmoid colon (arrow). **b.** Coronal fat-saturated post gadolinium-enhanced volumetric interpolated breath-hold examination (VIBE) image from MRE shows hyperenhancement in the inflamed, thickened sigmoid colon (arrow). **c.** Colonoscopic image of the sigmoid colon shows active inflammation, as evidenced by mucosal granularity, loss of normal vascular pattern, and ulcerations (arrows)

The results of the multivariate linear regression indicated that MaRIA scores (effect =1.54, *P*-value < 0.0001) and CDEIS (effect = 2.23, *p*-value < 0.0001) had a significant and positive association with FCP levels (Table 5, Fig. 5) while the R-square (0.68) specified the goodness of the model’s fit.

**Table 5:**
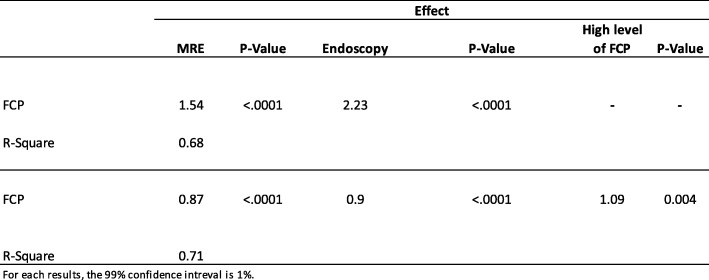
Multivariable analysis of FCP with MaRIA and CDEIS

## Discussion

This investigation shows that FCP correlates with validated MRE and colonoscopic scoring systems in a large prospective cohort of patients with colonic Crohn’s Disease. This data indicates that all three biometric tests provide clinically and morphologically relevant data that significantly correlate with disease activity. Positive correlations were observed between FCP and colonoscopy, FCP and MRE, and MRE and colonoscopy in univariate analyses and between FCP, colonoscopy, and MRE in multivariate analysis. MaRIA and CDEIS scores significantly correlated and the median levels of elevated FCP levels correspondingly rose with the severity of inflammation. It is beyond the scope of this study to consider which of these tests best or most accurately detects mucosal ulceration in the colon. As mentioned above, FCP, MRE and colonoscopy are highly correlated and complementary to each other; it may be a matter of preference, convenience, and cost-efficacy as to decide what particular modality to use for patient management. Moreover, we neither endorse that FCP is a total surrogate marker for colonoscopic or transmural disease activity in colonic CD nor that a single FCP measurement is sufficient for precise evaluation of colonic mucosal disease activity. The focus of our study was to demonstrate statistically the complementarity of FCP as a biomarker in colonic CD with colonoscopy and MRE.

The current set of investigations on the assessment of colonic CD with biomarkers and conventional tests continue to grow. Arai et al., Cerillo et al., and Ye at al. all showed similar concordance between FCP, MRE, and Crohn’s Disease activity but in the small bowel, not the colon, as in this study [9–11]. Nevertheless, these studies showed similar results and concordance. Our study can be added to the contemporary data that shows all three tests can be used, in a complementary fashion, to assess disease activity patients with CD now both in the small bowel and in the colon.

Multiple investigators have explored the range of FCP levels which most accurately reflects mucosal inflammation that range from 50 to 250 μg/mL [6–11]. While several prior studies have used FCP levels below 250 μg/mL [6–10], we confirmed that the FCP cutoff value of 250 μg/mL significantly correlated with the presence of active disease and severity of inflammation confirmed by both MRE and colonoscopy, respectively. This FCP cutoff value, which is in line with the Colombel et al. investigation, can be used to streamline testing [12].

We compared how a non-invasive biomarker: FCP correlates with conventional diagnostic modalities: colonoscopy and MRE in the assessment of disease activity. Beyond the degree of disease activity, assessment of mucosal healing is beyond the scope of this investigation. Clinical symptoms in combination with these tests contribute to successful disease control and monitoring response to treatment. A recent, large retrospective investigation by Kennedy et al. showed that FCP levels can be used to accurately monitor healing of mucosal inflammation in both the small bowel and colon; however, correlation with colonoscopy and MRE was not performed [14]. Ordas et al. showed that colonoscopy and MRE accurately assess healing of mucosal inflammation in the colon however FCP levels were not correlated [15]. Now that different investigators have shown each of these tests can be independently used to monitor healing and response to treatment, a future direction of research could be to prospectively examine healing and therapeutic response in patients with all three tests.

Our study has objective strength. We examined a large number of patients with biopsy-proven colonic CD. These patients were examined using a prospective study design and their tests were interpreted validated scoring systems. Our patients were referred from gastroenterologists and therefore subject to intrinsic referral bias however this bias was abated by the blinding the interpreting radiologists to the indication for the MRE exams. There were additional limitations to this investigation. This study was a single-institution experience at a tertiary academic center with gastroenterological and MRE availability and experience. Thus, our performance may not be transferrable to all populations due to accessibility and contraindications. If a patient has a contraindication to receiving procedural anesthetic sedation, then a patient cannot undergo colonoscopy. Certain patients may have contraindications to undergo MR imaging, such as medical devices, hardware, claustrophobia, or allergy to gadolinium contrast. FCP may not be a test that is widely available to all patients and may have substantial within-day variability [16]. Another limitation is the lack of validation of the results of our study in different clinical settings or different cohorts. It has been established that the diagnostic accuracies of FCP in colonic CD are generally considered to be different than those of small bowel CD; mucosal inflammation in the colon elevates FCP levels higher than mucosal inflammation in the small bowel [7–12, 14, 15].

The future research should be directed towards streamlining the schedule and clinical decision of when to perform these exams. Several studies have shown that these tests can be independently used to monitor mucosal healing and response to treatment, however never altogether. A potential avenue of future research may be to prospectively examine if all three tests provide statistically significant, congruent results that reflect mucosal healing and response to therapeutic modification in the same patient cohort.

## Conclusions

This investigation prospectively examines the relationship between FCP levels, MRE, and colonoscopy in a large cohort of patients with colonic CD. We showed that FCP significantly correlates with the degree of colonic inflammatory activity using validated MRE and colonoscopic scoring systems. Because all three biometric tests statistically correlate and produce reliably congruent results, then patients may be able avoid one or more difficult or contraindicated tests in favor of a more viable and equally efficacious test. The data from this investigation indicates that all three biometric tests provide clinically and morphologically relevant data that significantly correlate with disease activity. Positive correlations were observed between FCP and colonoscopy, FCP and MRE, and MRE and colonoscopy in univariate analyses and between FCP, colonoscopy, and MRE in multivariate analyses. Improved clinical results may be achieved by adopting a clinical strategy that relies on FCP, colonoscopy, and MRE together to then streamline monitoring and adjustment of therapy to reduce morbidity and improve long term outcomes.

## Data Availability

The datasets used and/or analyzed during the current study are available from the corresponding author on reasonable request.

## Abbreviations

CD: Crohn’s disease
CDAI: Crohn’s disease activity index
CDEIS: Crohn’s disease endoscopic index of severity
FCP: Fecal calprotectin
HIPAA: Health information portability and accountability act
MaRIA: Magnetic resonance index of activity
MRE: Magnetic resonance enterography
